# Serial Diurnal Salivary Cortisol Profiles in 667 Pregnant Women—Association With Cardiometabolic Complications

**DOI:** 10.1210/clinem/dgae202

**Published:** 2024-03-29

**Authors:** Alicia M Schowe, Darina Czamara, Marius Lahti-Pulkkinen, Polina Girchenko, Águeda Castro-Quintas, Lourdes Fañanas, Elisabeth B Binder, Katri Räikkönen

**Affiliations:** Department of Genes and Environment, Max Planck Institute of Psychiatry, 80804 Munich, Germany; Graduate School of Systemic Neuroscience, Ludwig-Maximilian-Universität, 80804 Munich, Germany; Department of Genes and Environment, Max Planck Institute of Psychiatry, 80804 Munich, Germany; Department of Psychology and Logopedics, University of Helsinki, 00014 Helsinki, Finland; Department of Psychology and Logopedics, University of Helsinki, 00014 Helsinki, Finland; Department of Evolutionary Biology, Ecology and Environmental Sciences (BEECA), Faculty of Biology, University of Barcelona, Institute of Biomedicine of the University of Barcelona (IBUB), 08007 Barcelona, Spain; Network Centre for Biomedical Research in Mental Health (CIBER of Mental Health, CIBERSAM), Institute of Health Carlos III, 28029 Madrid, Spain; Department of Evolutionary Biology, Ecology and Environmental Sciences (BEECA), Faculty of Biology, University of Barcelona, Institute of Biomedicine of the University of Barcelona (IBUB), 08007 Barcelona, Spain; Network Centre for Biomedical Research in Mental Health (CIBER of Mental Health, CIBERSAM), Institute of Health Carlos III, 28029 Madrid, Spain; Department of Genes and Environment, Max Planck Institute of Psychiatry, 80804 Munich, Germany; Department of Psychology and Logopedics, University of Helsinki, 00014 Helsinki, Finland; Department of Obstetrics and Gynecology, HUS Helsinki University Hospital, 00260 Helsinki, Finland

**Keywords:** pregnancy, cardiometabolic disorders, body mass index, hypothalamic-pituitary-adrenal (HPA) axis, cortisol awakening response, diurnal cortisol slope

## Abstract

**Context:**

Maternal obesity, hypertensive pregnancy disorders, and gestational diabetes (GDM) are linked to an increased risk of negative offspring health outcomes. This association may be mediated by maternal hypothalamic-pituitary-adrenal axis (HPA axis) activity, resulting in elevated maternal cortisol levels and fetal exposure, but evidence remains scarce.

**Objective:**

We (1) examined maternal diurnal cortisol profiles longitudinally across gestation, and (2) explored associations with maternal cardiometabolic complications.

**Methods:**

Women in the InTraUterine sampling in early pregnancy (ITU) study (n = 667) provided 7 salivary cortisol samples from awakening to bedtime up to 3 times during pregnancy (median gestational week 19.3, 25.7, and 38.1; n = 9356 samples). Changes in cortisol awakening response (CAR) and diurnal slope (indicative of HPA axis activity) and their associations with maternal body mass index (BMI), hypertensive pregnancy disorders and GDM were examined using linear mixed models.

**Results:**

The CAR declined in 60% to 67% of women, and the diurnal slope attenuated from early to late pregnancy (*b* = 0.006; *P* = .001). Higher BMI was associated with less decline in CAR (b = 0.031; *P* = .0004) and less attenuation in diurnal slope from early to late pregnancy (b = −0.001; *P* = .006). Hypertensive pregnancy disorders and GDM were not significantly associated with diurnal cortisol profiles.

**Conclusion:**

The attenuation in CAR and diurnal slope support HPA axis hyporesponsivity during pregnancy. Less attenuation of both markers in women with a higher BMI may indicate reduced adaption of the HPA axis to pregnancy, presenting a mechanistic link to offspring health outcomes.

Accumulating evidence suggests that maternal prepregnancy obesity, hypertensive pregnancy disorders, and gestational diabetes mellitus (GDM) are associated with poorer physical and mental health in the offspring later in life (1-6). These findings are compatible with the Developmental Origins of Health and Disease (DOHaD) framework (7). One frequently proposed mechanism underpinning these associations is altered fetal exposure to maternal circulating cortisol (8-10). Cortisol, which is the end product of the hypothalamic-pituitary-adrenal (HPA) axis, is released in response to physiological or psychosocial stress (11) and follows a diurnal pattern characterized by a sharp postawakening surge in concentration (the cortisol awakening response [CAR]) followed by a decline until bedtime (the diurnal slope) (12-14). Deviations from this typical diurnal pattern are thought to indicate HPA-axis dysregulation and have been linked to offspring birth outcomes such as length of gestation (15, 16) and birth weight (5, 17, 18).

Across gestation, maternal cortisol levels rise 2- to 4-fold, reflecting in part a positive feed-forward loop between placental corticotropin-releasing hormone production and maternal adrenal cortisol release (11, 19). While the diurnal cortisol pattern is maintained during pregnancy (11, 20, 21), the transient maternal hypercortisolemic state has been associated with attenuation in the CAR (15, 22) and the diurnal slope (23) with some studies suggesting that the CAR may become blunted as the pregnancy progresses (24-27). These normative pregnancy stage–specific changes are important to consider as they may alter associations with maternal cardiometabolic complications during pregnancy.

However, research that has examined associations between maternal cardiometabolic complications and diurnal cortisol profiles remains scarce. Existing studies are limited to studying maternal prepregnancy body mass index (BMI) (28, 29) or severe obesity (BMI ≥ 40) (5, 30), and we are aware of only one study with a focus on preeclampsia (31). Findings from these studies are mixed. While maternal prepregnancy BMI was not associated with the CAR or the diurnal slope (28, 29) and preeclampsia was not associated with the diurnal slope measured in mid/late pregnancy (31), maternal severe prepregnancy obesity was associated with a blunted CAR in early pregnancy (5). The mixed pattern of the findings may reflect the aforementioned differences in the gestational timing of measuring diurnal cortisol profiles. Furthermore, in none of the studies were diurnal cortisol profiles measured from early to late pregnancy. Hence these studies have not allowed the study of associations with change in the diurnal cortisol patterns along the course of pregnancy. In addition, in some of the studies the salivary sampling protocols have not allowed the study of the CAR (eg, saliva has been sampled at fixed bihourly time points starting at 8 Am (31) or on awakening and up to 1.5 hours thereafter (28)) or the diurnal slope (eg, saliva has been sampled only in the morning (5)). Moreover, in 2 of the studies, the sample sizes have been small (eg, 52 mothers in the study on severe obesity (5), 18 mothers in the study of preeclampsia (31)), limiting the external validity of the findings.

Against this background, the aim of our study was (1) to examine maternal diurnal cortisol profiles in early, mid, and late pregnancy in a large longitudinal study of 667 pregnant women across a total of 9356 diurnal salivary cortisol samples, and (2) to explore associations of maternal diurnal cortisol profiles across pregnancy with maternal prepregnancy BMI, hypertensive (chronic and gestational hypertension and preeclampsia) pregnancy disorders, and GDM.

## Materials and Methods

### Study Cohort

**Figure 1.**
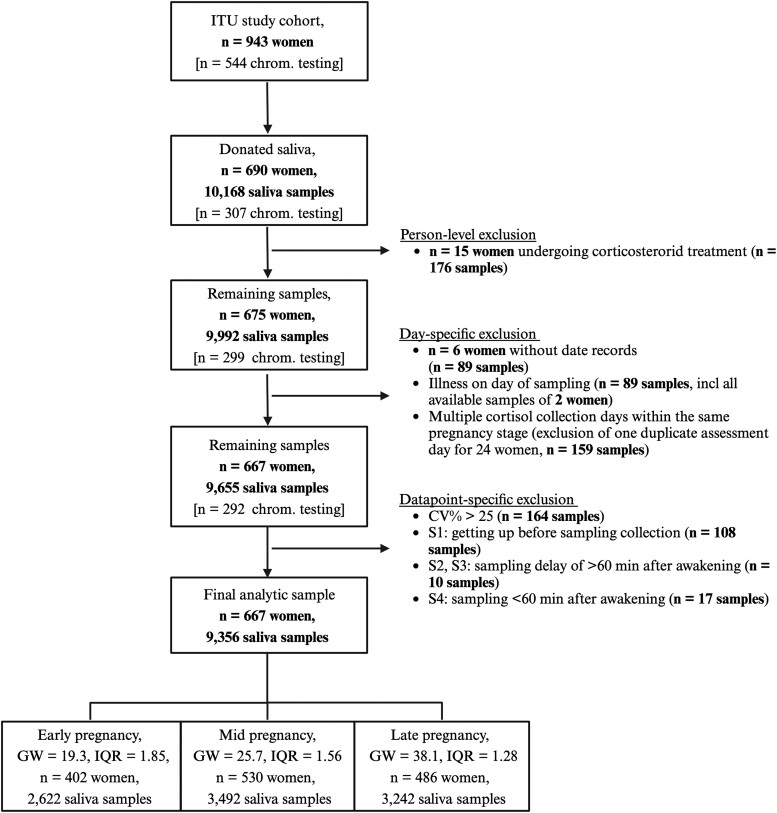
Flowchart of the exclusion criteria and salivary quality control steps applied to select our final analytic sample. As illustrated, we preprocessed the serial salivary cortisol data at several levels, applying exclusions criteria at the person level (eg, corticosteroid treatment), day level (eg, illness on a specific sampling day), and the individual data point–specific level (eg, sampling delay after awakening). CV%, coefficient of variation percentage; GW, median gestational week. Created with BioRender.com.

The INTraUterine Sampling in early pregnancy (ITU) study comprises 943 women and their singleton children born alive in Finland between 2012 and 2017 (see Kvist et al (32) for a detailed overview of the study design, recruitment, and sampling procedures). Briefly, women were recruited at the voluntary national 21-trisomy screen, offered free of charge to all pregnant women in Finland at gestational weeks (GWs) 9 to 21. Of these women, 544 (57.7%) were referred for fetal chromosomal testing at the Helsinki and Uusimaa Hospital District Fetomaternal Medical Center and thereafter cleared for fetal chromosomal abnormality. The rest, 399 (42.3%) women, had a negative 21-trisomy screen result and were not referred for fetal chromosomal testing. A flowchart of the sample selection for the present study is provided in Fig. 1. In total, 690 women (307 undergoing and 383 not undergoing chromosomal testing) donated diurnal saliva samples in early (<22 weeks), mid- (22-35 weeks), and late pregnancy (≥36 weeks) for cortisol assessment. Of these, 15 were excluded due to corticosteroid treatment (33), 6 due to missing date records, and 2 due to illness on the date of cortisol assessment, leaving 667 women with 9356 salivary cortisol samples in the analytic sample (see Fig. 1).

In comparison to women who did not provide salivary cortisol samples in the ITU cohort, women with data on salivary cortisol were less often referred for testing of fetal chromosomal abnormality, they less often had a secondary and more often upper tertiary education, less often smoked throughout pregnancy, had a lower prepregnancy BMI, and less often preeclampsia (Supplementary Table S1 (34)).

The ITU study was conducted according to the World Medical Association's Declaration of Helsinki, and the research protocol was approved by the ethics committee of the Helsinki and Uusimaa Hospital District (approval date January 6, 2015; reference No 269/13/03/00/09). All women provided written informed consent. In compliance with the General Data Protection Regulation of the European Union, the personal data of all participants were deidentified, protected at all times, and confidentiality agreements were signed by all personnel with ITU data access. Register data were obtained with permission from the register authority.

### Maternal Cardiometabolic Complications

We identified maternal cardiometabolic complications during pregnancy from medical records, the Medical Birth Register, and the Care Register for Health Care (HILMO). Maternal prepregnancy BMI was calculated from weight and height (kg)/height^2 (m^2) verified by measurement in the first antenatal clinic visit between 7 and 10 GWs. Hypertensive pregnancy disorders included gestational and chronic hypertension and preeclampsia (International Statistical Classification of Diseases and Health-Related Problems, 10th revision [ICD-10 codes I10-I15, O10-011, O13-O16; any/none]) and diabetes included GDM (ICD-10 code: O24.4; any/none). The diagnostic criteria for each cardiometabolic complication are provided in the Supplementary Table S2 (34).

### Diurnal Salivary Cortisol

Salivary cortisol sample collection, storage, and competitive enzyme immunoassay (enzyme-linked immunosorbent assay [ELISA]), kit (RE52611, TECAN, IBL, RRID:AB_3064818) have been previously described (32). The standard range is 0.015 to 3.0 µg/dL. The analytical sensitivity (limit of quantitation) is at 0.003 µg/dL, the 2 SD functional sensitivity at 0.005 µg/dL (0.05 in µg/L), and the mean concentration less than 20% (cross-reactivity of other tested substances <0.01%; intra-assay 3.2-6.1 µg/dL; interlot 4.2-17.0 µg/dL). The mean coefficient of variation across all samples measured in duplicate was 0.08%. To be consistent with the clinical use, we report all cortisol concentrations in µg/L. Mothers collected saliva using Salivette synthetic swabs in consecutively numbered tubes in early (<22 weeks, median = 19.3, IQR = 1.85), mid- (22-35 weeks, median = 25.7, IQR = 1.56), and late pregnancy (≥36 weeks, median 38.1, IQR = 1.28). The sampling took place when waking up (S1), 15 (S2) and 30 minutes (S3) thereafter; at 1030h (S4), 1200h (S5), 1700h (S6), and when going to sleep (“lights out”). The mothers were instructed to avoid brushing their teeth, eating, and drinking caffeinated products within 30 minutes before sampling. They were also asked to record the date and time at sample collection.

Our initial sample included 690 women with salivary samples (see Fig. 1). After excluding the 15 women under corticosteroid treatment, the sample that underwent quality control included 675 women and 9992 samples. Before the quality control, cortisol values below the lower limit of the assay range were truncated at 0.05 µg/L (2.4%, 240/9992, no values fell above the upper limit). For 24 women, multiple assessment days fell into the same pregnancy stage, and the samples of the day with the least complete observations, protocol adherence, or presence of day-specific outliers (ie, >2 SD of the day-specific mean) were excluded (1.6%, 159/9992). For 6 women, date records were missing, and their samples were excluded (0.9%, 89/9992). Further exclusion criteria were self-reported illness the day of sampling (0.9%, 89/99 992 samples, excluding of all samples of 2 women), getting up before the S1 sampling (1.1%, 108/9992 samples), sampling of S2 or S3 more than 60 minutes after awakening (0.1%, 10/9992 samples), S4 sampling within the first 60 minutes after awakening (0.2%, 17/9992 samples), or coefficient of variation greater than 0.25 (1.6%, 164/9992). Samples measured in duplicate were averaged. Missing sampling times (0.1%, 19/9992) were imputed according to sampling protocol for S2 to S6 or sample median for S1 and S7. The distribution of the cortisol samples was positively skewed, and thus natural log + 1 transformed (12, 35). To further reduce skewness, outliers (ie, >4 SD pregnancy stage-specific mean) were winsorized (9 samples).

The final analytic sample included 667 women (98.8% out of 675) and 9356 samples (93.6% out of 9992). Of these, 256 women provided salivary samples for all 3 pregnancy stages, 239 for 2 pregnancy stages, and 172 for 1 pregnancy stage. On average, 14 of the 21 samples were available for each woman (range, 3-21 samples; on average 6.5, 6.6, and 6.7 samples out of 7 samples in early, mid, and late pregnancy, respectively, with a minimum of 2 samples per pregnancy stage).

### Covariates

These included maternal age at delivery (years), parity (nulliparous/multiparous), and smoking throughout pregnancy (yes/no), which were derived from the Medical Birth Register, and whether or not the mother was referred for fetal chromosomal testing, which was embedded in the study design. Maternal mental and behavioral disorder diagnoses before or during pregnancy were derived from HILMO and included all primary and subsidiary diagnoses of all maternal hospital treatments since 1969 and of all outpatient treatments in public specialized medical care since 1998. The diagnoses are classified using the International Classification of Diseases 8th (ICD-8; codes 290-315) and 9th (ICD-9; codes 290-319) Revisions and the ICD-10 (codes F00-F99). Maternal education (secondary/lower tertiary/upper tertiary) was self-reported in early pregnancy. Timing-related covariates included time at awakening as absolute hours in decimals (eg, 08h45 = 8.75h), and season (winter [October to March] vs summer [April to September] months) (14, 36).

### Statistical Analyses

All analyses were carried out using R and its packages (R version 4.1.3) (37). The analysis code is publicly available at https://github.com/aschowe/ITU_cortisol_2023.git. The 2 main components of the diurnal cortisol rhythm, the CAR, and the diurnal slope were analyzed in 2 separate analyses using linear mixed models (12, 23) (*nlme*-package, version 3.1 (38)). Linear mixed models allow the analysis of repeated measures through the inclusion of random intercepts and slopes. In addition, they permit explicit modeling of the variance-covariance structure to account for unequal time intervals in the repeated design and heterogeneous variation within occasions. In the morning-model, log-transformed cortisol concentrations from S1 to S3 from all pregnancy stages were used as the within-person dependent variable. In the diurnal slope-model, log-transformed cortisol concentrations from the morning peak (maximum of S1, S2, and S3) to S7 from all pregnancy stages were used as the within-person dependent variable.

#### Salivary cortisol profiles during pregnancy

To examine changes in the CAR and the diurnal slope across gestation, we first determined the best fitting mixed model structure using restricted maximum likelihood estimation (REML, see Supplementary Fig. S1 (34) and Supplementary Table S3 (34) and S4 (34)) (12). Time since awakening (ie, time at sampling centered at awakening) was included as a within-person fixed effect (ie, independent variable) in each model so that the intercept indicates the cortisol concentration at awakening, and the slope the change from this baseline (ie, representing the CAR in the morning model and the diurnal slope in the diurnal slope model). Both models included a quadratic effect of time (since awakening) to account for the potential curvilinear shape of change from baseline. A random intercept for ELISA analysis plate was added to account for batch effects. The nested, repeated-measures design (of repeated occasions within each pregnancy stage nested within each person) was modeled by including random intercepts for participant (participant ID) and pregnancy stage (early, mid, late pregnancy).

In subsequent models, we tested a random slope for time and a (heterogeneous) continuous autoregressive covariance structure of order 1 (AR1) would significantly improve model fit. By changing the variance-covariance structure to heterogeneous AR1, we allow varying variances and varying covariances between our irregularly spaced occasions instead of assuming equal variance and equal covariance across all occasions. The most parsimonious model with the significantly lowest Akaike information criterion (AIC) value was determined the best fit. For the fixed-effects structure of the final model, we kept only the timing-related covariates that were significantly associated with cortisol concentration in the model (**χ**^2^ test *P* < .05; see Supplementary Fig. S1 (34) and Supplementary Table S5 (34) and S6 (34)). In addition to studying the CAR and the diurnal slope in the mixed models, we describe the CAR and the total cortisol output using the area under the curve with respect to increase (AUC_i_) and ground (AUC_g_), respectively (35).

To estimate the stability of cortisol measures during pregnancy, we computed intraclass correlation coefficients (ICCs) based on the mixed-model variance decomposition of the morning and the diurnal model (see supplemental materials (34) for the detailed formula) (12). Specifically, we provide both the ICC for stability of cortisol measures within persons within pregnancy stages and the ICC for stability of cortisol measures within persons throughout pregnancy. A higher ICC indicates higher stability and greater correlation of cortisol measures within the same individual. Moreover, we computed ICCs to indicate the stability of the CAR in the form of the AUC_i_, the total cortisol output in the form of the AUC_g_, and the stability of individual cortisol samples (peak, S1-S7) within persons throughout pregnancy using the “icc” function of the *irr* package (version 0.84.1) (39).

#### Salivary cortisol profiles in relation to cardiometabolic complications

To study whether the CAR and the diurnal slope are associated with cardiometabolic complications, we proceeded as follows (see Supplementary Fig. S2 (34) for a visual representation): First, each cardiometabolic condition was added separately as a between-person fixed effect to the morning and to the diurnal model to test for significant main effects on cortisol concentration throughout pregnancy. Next, we included the interaction term of the given cardiometabolic complication and the within-person time slope to test for significant association with the CAR and diurnal slope throughout pregnancy. In the third step, we added a 3-way interaction term of cardiometabolic complication × within-person time × within-person pregnancy stage to the respective model to examine whether the change in CAR and diurnal slope from early to mid and late pregnancy was dependent on the given cardiometabolic complication. Lastly, we repeated each step including chromosomal testing group, maternal age, parity, smoking during pregnancy, lifetime mental disorders, and level of education as covariates. To facilitate interpretation, all continuous variables were centered at the grand mean and effect sizes expressed in percentage change [formula: (exp(*b*)−1) × 100] (23).

## Results

### Sample

**Figure 2.**
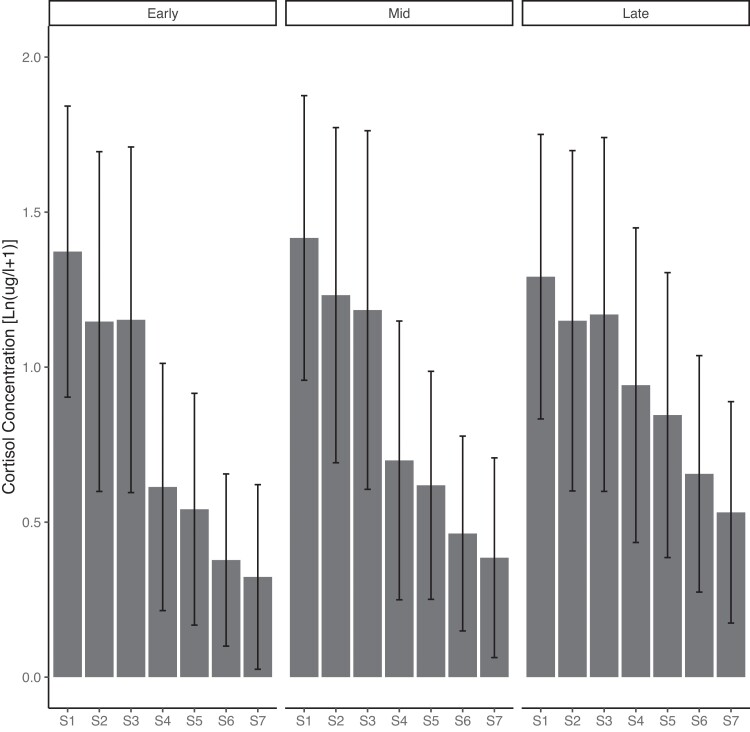
Bar graph of the log-transformed mean cortisol concentration by time of sampling and pregnancy stage. Early pregnancy: median gestational week = 19.3 (IQR = 1.85), midpregnancy: median gestational week = 25.7 (IQR = 1.56), late pregnancy: median gestational week = 38.1 (IQR = 1.28). S1 = awakening, S2 = awakening + 15 min, S3 = awakening + 30 min, S4 = 10:00, S5 = 12:00, S6 = 17:00, S7 = bedtime. Error bars indicate SD.

Sample characteristics are reported in Table 1. Table 2 shows the raw salivary cortisol concentrations and sampling times for each pregnancy stage. The log-transformed mean cortisol concentrations and their standard deviations are illustrated in Fig. 2. In addition, Table 3 shows the CAR calculated using the AUC_i_ and the total diurnal cortisol output calculated using the AUC_g_ at each pregnancy stage.

**Table 1.** Sample characteristics

Characteristic	Overall N = 667
Chromosomal testing (yes), No. %	292 (44%)
Maternal age, M (SD), y	34.63 (4.82)
Highest level of maternal education, No. %	
Secondary	122 (19%)
Lower tertiary	177 (27%)
Upper tertiary	356 (54%)
Parity (primiparous), No. %	338 (51%)
Maternal BMI, M (SD)	23.82 (3.93)
Hypertensive disorders (yes), No. %	35 (5.2%)
Gestational diabetes (yes), No. %	139 (20.8%)
Maternal lifetime mental disorder (yes), No. %	77 (12%)

Abbreviations: BMI, body mass index; M, mean; SD, standard deviation.

**Table 2.** Raw cortisol concentrations at each occasion by pregnancy stage

	Early pregnancy(GW = 19.3, IQR = 1.85)N = 402		Mid pregnancy(GW = 25.7, IQR = 1.56)N = 530		Late pregnancy(GW = 38.1, IQR = 1.28)N = 486		
Occasion	ConcentrationM (SD), μg/L	Time*^a^*,M (SD)	ConcentrationM (SD), μg/L	Time*^a^*,M (SD)	ConcentrationM (SD), μg/L	Time*^a^*,M (SD)	Cortisol ICC*^b^*
Morning peak, μg/L	4.18 (3.09)	7.32 (1.14)	4.38 (2.78)	7.36 (1.12)	3.77 (0.12)	7.79 (1.18)	0.30*^c^*
S1, μg/L	3.44 (2.56)	7.13 (1.15)	3.59 (2.47)	7.16 (1.11)	3.02 (1.84)	7.58 (1.17)	0.25*^c^*
S2, μg/L	2.71 (2.77)	7.40 (1.14)	2.96 (2.37)	7.41 (1.10)	2.65 (2.08)	7.83 (1.16)	0.21*^c^*
S3, μg/L	2.70 (2.37)	7.63 (1.13)	2.85 (2.32)	7.68 (1.11)	2.78 (2.27)	8.09 (1.15)	0.28*^c^*
S4, μg/L	1.02 (0.98)	10.57 (0.36)	1.28 (1.79)	10.63 (0.59)	1.94 (1.78)	10.61 (0.44)	0.07*^e^*
S5, μg/L	0.86 (0.89)	12.15 (0.47)	0.99 (0.85)	12.30 (0.77)	1.60 (1.36)	12.19 (0.58)	0.19*^c^*
S6, μg/L	0.53 (0.65)	17.23 (0.58)	0.69 (0.87)	17.23 (0.65)	1.10 (1.23)	17.20 (0.72)	0.05
S7, μg/L	0.49 (1.15)	22.99 (1.10)	0.58 (0.96)	23.02 (1.05)	0.84 (1.07)	23.31 (1.13)	0.13*^d^*

Abbreviations: AUC_i_, area under the curve with respect to increase; CAR, cortisol awakening response; GW, gestational week; ICC, intraclass-coefficient; IQR, interquartile range; M, mean.

^*a*^Time presented in hours in decimals (ie, 7.5 = 7:30 Am).

^*b*^Calculated in complete cases.

^*c*^Less than .0001. *^d^*Less than .01. *^e^*Less than .05.

**Table 3.** Cortisol summary indices by pregnancy stage

Parameter	Early pregnancy(GW = 19.3, IQR = 1.85) N = 402	Mid pregnancy (GW = 25.7, IQR = 1.56) N = 530	Late pregnancy (GW = 38.1, IQR = 1.28) N = 486	ICC*^a^*
Cortisol awakening response (AUC_i_, ln[μg/L + 1] from S1 to S3), M (SD)	−0.08 (0.18)	−0.08 (0.18)	−0.05 (0.16)	0.14*^c^*
Women with absent cortisol awakening response*^b^*, No. %	255 (67%)	339 (66%)	284 (60%)	
Total diurnal cortisol output (AUC_g_, ln[μg/L + 1] from S1 to S7), M (SD)	8.13 (4.00)	9.29 (4.37)	11.69 (5.30)	0.39*^c^*

Abbreviations: AUC_g_, area under the curve with respect to ground; AUC_i_, area under the curve with respect to increase; GW, gestational week; ICC, intraclass-coefficient; M, mean.

^*a*^Calculated in complete cases.

^*b*^Based on n_AUCi≤0_/n_total_.

^*c*^Less than .0001.

### Cortisol Awakening Response and Diurnal Slope During Pregnancy

Morning cortisol levels did not show the expected CAR (ie, the significant increase in cortisol concentration in the first 30-45 minutes after awakening; see Tables 2-4) as 67% (255/402), 66% (339/530), and 60% (284/486) of women presented with a declining CAR (see Table 3, based on N_(AUCi ≤ 0)_/N_total,_ and Supplementary Fig. S3 (34) for a histogram of the CAR) in early, mid, and late pregnancy, respectively. Of the women who provided salivary samples in at least 2 pregnancy stages (n = 495), 430 (86.8%) showed a declining CAR at least once, and 185 (37.3%) in all 3 pregnancy stages. Neither time at awakening nor season affected cortisol differently depending on pregnancy stage (see Supplementary Table S5 (34)) The 3 morning cortisol measurements were strongly correlated within pregnancy stages in the same individuals but showed poor to moderate stability within persons throughout pregnancy (see Tables 2 and 4) Average morning cortisol concentration in the morning model was highest in mid compared to early (see Table 4) and late pregnancy (mid > late pregnancy, b = −0.16, SE = 0.03; *P* < .0001). Average morning slope attenuated from early and mid to late pregnancy (see Table 4, time × mid pregnancy vs time × late pregnancy, b = 0.19, SE = 0.06; *P* = .002) consistent with a lower number of women with a declining CAR in late pregnancy (see Table 3).

**Table 4.** Final morning model^a^

Predictor	b	SE	t	*P*	Interpretation
Intercept	1.31	0.026	51.05	<.0001	Grand average cortisol at awakening in early pregnancy
Time	−0.709	0.074	−9.608	<.0001	−50.8% lower cortisol per h
Time squared	0.557	0.099	5.613	<.0001	Reversal of cortisol slope by 74.5%
Mid pregnancy (ref = early pregnancy)	0.077	0.029	2.625	.009	8.0% greater cortisol in mid pregnancy
Late pregnancy (ref = early pregnancy)	−0.038	0.03	−1.264	.207	
Time at awakening	1e-03	0.011	0.091	.927	
season (winter)	0.039	0.023	1.722	.086	
Time × mid pregnancy (ref = early pregnancy)	−0.018	0.066	−0.278	.781	
Time × late pregnancy (ref = early pregnancy)	0.168	0.067	2.505	.012	18.3% attenuated slope in late pregnancy
Random component	Variance component				
Var(b_i_) (ELISA plate)	0.001				
Var(c_i0j_)(person-level)	0.061				
Covariance	0.044				
Var(c_i1j_) (slopes)	0.274				
Var(d_ijk_) (pregstage level)	0.057				
var(*ε_ijk_*) (residual)	0.129				
ICC estimates					
ICC within person throughout pregnancy	0.12				Cortisol correlation of the same individual throughout pregnancy
ICC within person within pregnancy stage	0.75				Cortisol correlation of the same individual within pregnancy stages
N morning model	4047				
Total R explained	75.3				

Abbreviations: ELISA, enzyme-linked immunosorbent assay; ICC, intraclass coefficient; ref, reference; RI, random intercept; RS, random slope.

^*a*^Morning concentrations were modeled best with an AR(1) variance-covariance structure for 4 levels of nesting and a random slope for time since awakening (see Supplementary Table S3 (34)).

**Figure 3.**
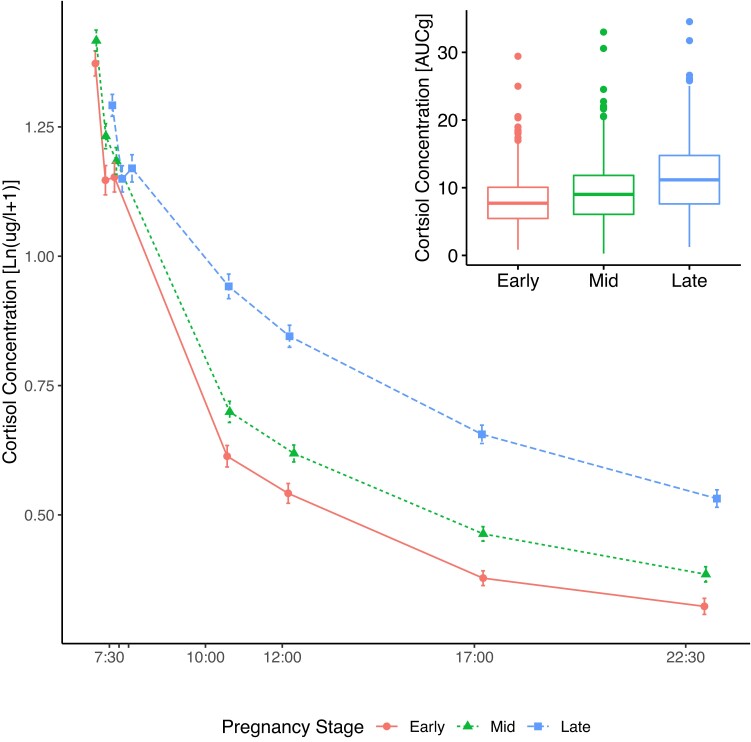
Diurnal cortisol profiles in early, mid, and late pregnancy from awakening to bedtime. Mean concentration (in log-transformed μg/L + 1) by mean time of day at sampling and pregnancy stage (early pregnancy = circles [median gestational week = 19.3, IQR = 1.85], midpregnancy = triangles [median gestational week = 25.7, IQR = 1.56], late pregnancy = squares [median gestational week = 38.1, IQR = 1.28]). Average time at awakening and subsequent sampling at +15 min and +30 min varied between pregnancy stages. The cortisol rise from early to mid and late pregnancy in total cortisol output is illustrated in the box plot in the upper right corner. AUCg, area under the curve with respect to ground. Error bars indicate the standard error.

Diurnal cortisol levels followed the expected decline from peak to bedtime with an overall increase of cortisol concentration of 10.0% and 15.7% from early to mid and late pregnancy, respectively (Fig. 3 and Table 5; mid < late pregnancy, b = 0.82, SE = 0.02; *P* = .0002). The diurnal slope was attenuated in late compared to early (see Table 5) and mid pregnancy (time × mid pregnancy vs time × late pregnancy, b = 0.006, SE = 0.002; *P* = .0003). Like in the morning model, the diurnal cortisol concentrations were more stable within pregnancy stages in the same individuals compared to the cortisol correlations within individuals throughout pregnancy (see Table 5). Total diurnal cortisol output calculated using the AUC_g_ presented as the most stable indicator of individual differences in cortisol concentration during pregnancy (see Table 3).

**Table 5.** Final diurnal model^a^

Predictor	b	SE	t	*P*	Interpretation
(Intercept)	1.297	0.022	57.662	<.0001	Grand average at awakening in early pregnancy
Time	−0.165	3e-03	−53.505	<.0001	−15.2% lower cortisol with every h
Time squared	0.006	2e-04	42.21	<.0001	0.6% attenuation in slope with every h
Mid pregnancy (ref = early pregnancy)	0.096	0.025	3.821	.0001	10.0% greater cortisol in mid pregnancy
Late pregnancy (ref = early pregnancy)	0.146	0.027	5.334	<.0001	15.7% greater cortisol in late pregnancy
Time at awakening	−0.059	0.016	−3.562	.0004	−5.7% lower cortisol with every h later from awakening
Season (winter)	0.02	0.024	0.812	.417	
Time × mid pregnancy (ref = early pregnancy)	−5e-04	2e-03	−0.263	.793	
Time × late pregnancy (ref = early pregnancy)	0.006	2e-03	3.183	.001	0.7% attenuated decline with every h from awakening in late pregnancy
Time × time at awakening	5e-03	1e-03	3.901	<.0001	0.5% attenuated decline with every h from awakening
Mid pregnancy × season (winter)	−0.01	0.03	−0.334	.739	
Late pregnancy × season (winter)	0.072	0.036	2.038	.042	7.5% greater cortisol increase
Mid pregnancy × time at awakening	−3e-03	0.021	−0.127	.899	
Late pregnancy × time at awakening	0.014	0.022	0.623	.533	
Time × mid pregnancy × time at awakening	1e-04	2e-03	0.072	.942	
Time × late pregnancy × time at awakening	−4e-03	2e-03	−2.118	.034	−0.3% less attenuation in decline with every h from awakening in late compared to early pregnancy
Random component	Variance component				
Var(b_i_) (ELISA plate)	0.001				
Var(c_i0j_) (person-level)	0.048				
Covariance	−0.75				
Var(c_i1j_) (slopes)	0.008				
Var(d_ijk_) (pregstage-level)	0.019				
var(*ε_ijk_*) (residual)	0.189				
ICC estimates					
ICC within persons throughout pregnancy	0.18				Estimated cortisol correlation of the same individual throughout pregnancy
ICC within persons within pregnancy stages	0.26				Estimated cortisol correlation of the same individual within pregnancy stages
N diurnal model	6687				
Total R explained	26.0				

Abbreviations: ELISA, enzyme-linked immunosorbent assay; ICC, intraclass coefficient; RI, random intercept; RS, random slope.

^*a*^Diurnal cortisol concentrations were modeled best with a heterogeneous AR(1) variance-covariance structure for 4 levels of nesting and a random slope for time since awakening (see Supplementary Table S4 (34)).

### Cortisol Awakening Response and Diurnal Slope in Relation to Maternal Cardiometabolic Complications

**Figure 4.**
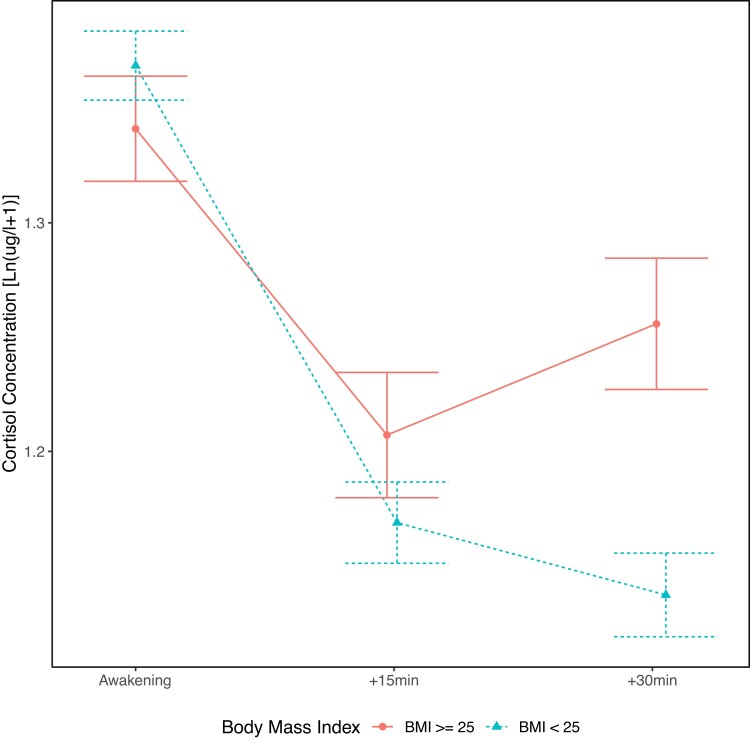
Body mass index (BMI)-related morning cortisol changes during pregnancy. Mean cortisol concentration (in log-transformed μg/L + 1) at awakening, and +15 minutes and +30 minutes post awakening are shown for women with BMI greater than or equal to 25 (circles) and BMI less than 25 (triangles), illustrating the cortisol awakening response declines less in women with higher BMI in the morning model across all pregnancy stages. Error bars indicate the standard error.

**Figure 5.**
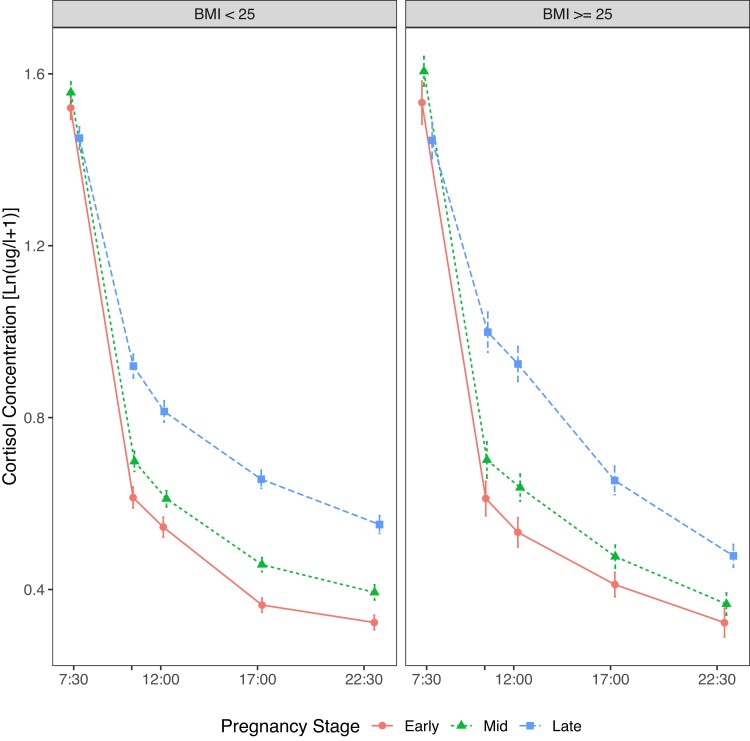
Body mass index (BMI)-related diurnal cortisol changes during pregnancy. Mean cortisol concentration (in log-transformed μg/L + 1) from the diurnal peak to bedtime in early (circles, median gestational week = 19.3, IQR = 1.85), mid (triangles, median gestational week = 25.7, IQR = 1.56), and late (squares, median gestational week = 25.7, IQR = 1.56) pregnancy is shown for women with BMI less than 25 (left) and BMI greater than or equal to 25 (right), illustrating the association between higher BMI and attenuated diurnal slope from early to late pregnancy in the diurnal model. Error bars indicate the standard error.

Higher maternal prepregnancy BMI was associated with less decline in the CAR independent of pregnancy stage and covariates (see Table 6, Supplementary Table S7 (34), and Fig. 4) and less attenuation in the diurnal slope from early to late pregnancy (Table 7 and Fig. 5; change in diurnal slope from mid to late pregnancy was not significantly affected, see Supplementary Table S8 (34)). Further examination of these significant 2- and 3-way interaction results using stratified analyses for women with a BMI greater than or equal to 25 and BMI less than 25 confirmed less decline in the CAR (CAR in the high BMI group: b = −0.40; *P* = .002 vs in low BMI: b = −0.834; *P* < .0001) and less attenuation in diurnal slope from early to late pregnancy (change in diurnal slope from early to late pregnancy in the high BMI group: b = 0.007; *P* = .09 vs in low BMI: b = 0.012; *P* < .0001) in women with a higher BMI. The cortisol means and SEs on each occasion by BMI (BMI ≥ 25 and BMI < 25) and pregnancy stage are provided in Supplementary Table S9 (34). Hypertensive pregnancy disorders and GDM were not associated with the CAR or diurnal slope (see Tables 6 and 7, Supplementary Table S7 (34) and S8 (34)).

**Table 6.** Morning model: associations with cardiometabolic complications

	Model 1*^a^*				Model 2*^b^*				
Predictor	b	SE	t	*P*	b	SE	t	*P*	Interpretation
Step 1: testing for main effects on cortisol concentration									
Maternal hypertensive disorder (yes)	−0.05	0.065	−0.769	.442	−0.022	0.067	−0.326	.745	
GDM (yes)	0.027	0.036	0.754	.451	0.042	0.036	1.179	.239	
Maternal BMI	−2e-04	4e-03	−0.045	.964	2e-03	4e-03	0.473	.636	
Step 2: testing for effects on CAR throughout pregnancy									
Maternal hypertensive disorder (yes) × time	0.048	0.152	0.318	.75	−3e-03	0.158	−0.016	.987	
GDM (yes) × time	0.173	0.082	2.099	.036	0.15	0.083	1.802	.072	
Maternal BMI × time	0.031	0.008	3.693	2e-04	0.03	0.009	3.542	4e-04	Every unit greater BMI is associated with 3.1% less decline in CAR
Step 3: testing for effects on change in CAR from early to mid and late pregnancy									
Maternal hypertensive disorder (yes) × time × mid pregnancy (ref = early pregnancy)	0.26	0.328	0.792	.429	0.2	0.334	0.598	.55	
Maternal hypertensive disorder (yes) × time × late pregnancy (ref = early pregnancy)	−0.201	0.337	−0.597	.55	−0.216	0.34	−0.637	.524	
GDM (yes)× time × mid pregnancy (ref = early pregnancy)	0.04	0.163	0.247	.805	0.04	0.164	0.243	.808	
GDM (yes)× time × late pregnancy (ref = early pregnancy)	−0.01	0.17	−0.058	.954	0.012	0.171	0.071	.944	
Maternal BMI × time × mid pregnancy (ref = early pregnancy)	−0.018	0.016	−1.101	.271	−0.025	0.016	−1.545	.122	
Maternal BMI × time × late pregnancy (ref = early pregnancy)	−0.005	0.017	−0.313	.754	−0.007	0.017	−0.379	.704	

Maternal BMI, hypertensive disorder, and GDM tested in separate models.

Abbreviations: BMI, body mass index; CAR, cortisol awakening response; GDM, gestational diabetes mellitus; ref, reference.

^*a*^Model 1: full morning model (fixed effects): logCort_S1-S3_ = time + time^2 + pregnancy stage + time at awakening + season + time × pregnancy stage.

^*b*^Model 2: M1 + maternal age + level of highest education + parity + smoking throughout pregnancy + chromosomal testing + lifetime mental disorder diagnosis.

**Table 7.** Diurnal model: associations with cardiometabolic complications

	Model 1*^a^*				Model 2*^b^*				
Predictor	b	SE	T	*P*	b	SE	t	*P*	Interpretation
Step 1: testing for main effects on cortisol concentration									
Maternal hypertensive disorder (yes)	−0.008	0.04	−0.196	.845	−0.009	0.041	−0.226	.821	
GDM (yes)	−0.0004	0.022	−0.011	.991	0.007	0.022	0.327	.744	
Maternal BMI	−0.0003	2e-03	−0.138	.89	3e-04	2e-03	0.149	.882	
Step 2: testing for effects on diurnal slope throughout pregnancy									
Maternal hypertensive disorder (yes) × time	−0.001	4e-03	−0.379	.704	−0.0009	0.004	−0.239	.811	
GDM (yes) × time	−0.003	2e-03	−1.619	.105	−0.003	0.003	−1.413	.158	
Maternal BMI × time	−0.0004	2e-04	−1.807	.071	−0.0003	0.0002	−1.6	.11	
Step 3: testing for effects on change in diurnal slope from early to mid and late pregnancy									
Maternal hypertensive disorder (yes) × time × mid pregnancy (ref = early pregnancy)	0.003	0.008	0.374	.708	0.002	0.008	0.27	.787	
Maternal hypertensive disorder (yes) × time × late pregnancy (ref = early pregnancy)	−0.0006	0.009	−0.065	.948	−0.0006	0.009	−0.069	.945	
GDM (yes) × time × mid pregnancy (ref = early pregnancy)	0.002	0.004	0.48	.631	0.002	4e-03	0.432	.665	
GDM (yes) × time × late pregnancy (ref = early pregnancy)	0.003	0.005	0.607	.544	0.002	5e-03	0.473	.636	
Maternal BMI × time × mid pregnancy (ref = early pregnancy)	−0.0006	0.0004	−1.444	.149	−0.0006	4e-04	−1.429	.153	
Maternal BMI × time × late pregnancy (ref = early pregnancy)	−0.001	5e-04	−2.716	.007	−1e-03	5e-04	−2.764	.006	0.1% reduced attenuation in diurnal slope from early pregnancy to late pregnancy with every unit greater BMI

Maternal BMI, hypertensive disorder, and GDM tested in separate models.

Abbreviations: BMI, body mass index; GDM, gestational diabetes mellitus; ref, reference.

^*a*^Model 1: full diurnal model (fixed effects): logCort_peak-S7_ = time + time^2 + pregnancy stage + time at awakening + season + time × pregnancy stage + time × time at awakening + season × pregnancy stage + time × time at awakening × pregnancy stage.

^*b*^Model 2: M1 + maternal age + level of highest education + parity + smoking throughout pregnancy + chromosomal testing + lifetime mental disorder diagnosis.

### Exploratory Post Hoc Analyses

The low frequency of the expected CAR pattern prompted several post hoc follow-up analyses. First, comparing women with and without the expected CAR showed that S1 cortisol concentration was on average higher and more frequently the highest value of the day in women without the expected CAR (see Supplementary Tables S10-S12 (34)). Moreover, women with the expected CAR in mid and late pregnancy were slightly younger and, in late pregnancy, less often referred for fetal chromosomal abnormality testing, more often primiparous, and had a higher level of education. Second, CAR patterns were examined in 2 additional samples, in the Arvo Ylppö Longitudinal Study (AYLS) including 446 nonpregnant young adult women (40) and in the Intramural Maternal Epi-Project including 112 pregnant women (see the supplementary materials (34) for more information on the study cohorts and salivary cortisol collection (41)). Using the same salivary collection protocol as used in this study, the expected CAR pattern was absent in 17.9% (n = 366/446) of women in the AYLS cohort (mean AUC_i_ = 1.18, SD = 3.24, Supplementary Table S13 (34)), which is consistent with studies of the general population including objective time records (42). In the Intramural Maternal Epi-Project pregnancy cohort, the expected CAR (45 min postawakening minus awakening concentration) was absent in 30.8% (28/91; mean = 0.13, SD = 0.22), 41.5% (39/94; mean = 0.05, SD = 0.21), and 33.7% (ie, 28/83; mean = 0.06, SD = 0.20) in early (median GW = 14.43, interquartile range [IQR] = 5.57), mid (median GW = 24.71, IQR = 8.57) and late pregnancy (median GW = 35.57, IQR = 3.43), indicating slightly higher absence rates compared to the general population (35, 42).

## Discussion

In this study, we examined maternal diurnal cortisol profiles in early, mid, and late pregnancy in a large longitudinal study of 667 pregnant women and explored the associations of cortisol profiles with multiple maternal cardiometabolic complications. Our longitudinal analysis revealed poor to modest stability of individual differences in cortisol concentration, frequent absence of the expected CAR pattern, and diurnal slope attenuation from early to late pregnancy. Moreover, a higher prepregnancy BMI (but not hypertensive pregnancy disorders or GDM) resulted in an altered CAR throughout pregnancy and reduced attenuation in diurnal slope from early to late pregnancy.

Following up on the frequent absence of the expected CAR pattern in all 3 pregnancy stages, we demonstrate the expected CAR in a sample of nonpregnant young adult women based on the same collection protocol, yet again frequent CAR decline in another pregnant population. Previous studies have found a CAR similar to nonpregnant samples (20, 21, 23, 28). However, consistent with our findings, there are also reports of a mean decline in the CAR in GWs 19 (25), 21 (26), 24.8 (24), and 28 (27). Moreover, in our additional analyses, we show that there are only slight individual differences (eg, maternal age) between women with and without the expected CAR that cannot fully explain the declining CAR. Altogether, a shift toward a declining CAR may thus be a pregnancy characteristics indicative of a hyporesponsive HPA axis. On the other hand, it may also indicate morning state (eg, morning sickness (43), pregnancy-specific anxiety (44, 45), perceived emotional support (46)) and/or lifestyle (eg, disrupted sleep (27, 47)) changes that interfere with the assessment of the CAR, in particular, during pregnancy (48). These potential causes of a declining CAR require further investigation to improve CAR assessment and its interpretation in future studies of pregnant women.

Moreover, we show lower morning cortisol concentration in late pregnancy. This effect may be the result of a more relaxed morning routine (eg, later awakening, see Table 2) due to maternal leave from GW 37 or subsiding morning sickness (43), presenting one of the factors that require special consideration for CAR assessment during pregnancy. Likewise, seasonal variation in cortisol concentration found in the general population (14) was maintained and requires consideration when following pregnant women over several months of gestation. ITU cortisol concentration at awakening was similar to cortisol concentration in the Intramural Maternal Epi-Project (41) and reporting by Murphy et al (24) of cortisol for 220 women in GWs 14.1, 24.8, and 34.7. Furthermore, cortisol rise and diurnal slope attenuation in the diurnal model confirm previous reports of normative gestation-related changes, indicative of a blunting of diurnal HPA axis characteristics during pregnancy (23, 24). Likewise, the modest stability of individual differences in diurnal cortisol supports previous findings (24).

Maternal prepregnancy BMI, but not hypertensive pregnancy disorders or GDM, was associated with alterations both in the CAR and change in the diurnal slope across gestation. The positive association between the CAR and BMI is in contrast to previous studies showing lower +30-minute cortisol concentration in severely obese women in GWs 16 and 29 (5, 28) or no association with BMI (30), which may be explained by sample differences. Specifically, the severely obese women with GDM in the former study may differ from our largely normal to overweight sample in clinical and lifestyle characteristics (eg, sleep duration and quality (49)). For example, Bublitz et al (27) report that sleep apnea in obese women with GDM is prevalent and associated with a blunted CAR. In our study, the BMI-related reduced attenuation in diurnal slope, in combination with reduced decline in CAR, may indicate less adaption of the HPA axis to pregnancy. In line with one previous investigation (31), hypertensive pregnancy disorders were not associated with any cortisol parameter although this may be due to the small number of preeclampsia cases in both studies, which in our study represented the population prevalence (2%-3%). To the best of our knowledge, this is the first study to investigate the effect of GDM on salivary diurnal cortisol changes. Lack of association may indicate the need to consider alternative pathways underlying the associations with increased risk of physical and mental health adversities in offspring (50, 51). Yet again, obesity and GDM often co-occur (52) so that overweight and obese women with GDM may be affected by HPA-axis dysregulation in a complex interplay with other pathways (53, 54). However, this will require further investigation.

### Strength and Limitations

To the best of our knowledge, this study poses the most extensive longitudinal investigation of the diurnal cortisol patterns in pregnant women. It applied state-of-the-art multilevel analysis ensuring optimal use of all data points and allowing simultaneous analysis of change in cortisol concentration within days and across gestation. This longitudinal perspective resulted in cortisol feature and pregnancy stage–specific findings not possible to detect in cross-sectional designs. Moreover, BMI and cardiometabolic diagnoses were based on reliable nationwide health-care registers instead of self-reports. However, it is also limited by several factors. First, awakening and sampling times were self-reported and not objectively assessed (eg, via electronic monitoring caps). Moreover, cortisol samples were collected after GW 12 so that associations of the CAR and diurnal slope with cardiometabolic conditions could not be tested for the first trimester. Next, given the focus on cardiometabolic disorders, this study did not include momentary or day-specific psychological well-being, which may explain more cortisol variation (38). Likewise, assessment of sleep quality and quantity the night before morning cortisol collection may have affected our CAR assessment and poses an important factor to consider in future studies (20). Lastly, participants in our sample were recruited from the Helsinki and Uusimaa Hospital District area in Finland, representing a Nordic, high-income population, limiting the generalizability of our findings to other populations.

### Conclusions

The decline in the CAR and attenuation in diurnal slope supports normative HPA axis hyporesponsivity during pregnancy. Higher prepregnancy BMI is associated with less decline in CAR regardless of pregnancy stage and resulted in less attenuation in the diurnal slope from early to late pregnancy. Less attenuation of both of these markers may indicate reduced adaption of the HPA axis to pregnancy, presenting a mechanistic link to offspring health outcomes.

## Data Availability

Data sets generated during the current study are not publicly available but will be made available on reasonable request. Requests are subject to further review by the national register authority and by the ethical committees.

## Abbreviations

AUC_g_: area under the curve with respect to ground
AUC_i_: area under the curve with respect to increase
BMI: body mass index
CAR: cortisol awakening response
ELISA: enzyme-linked immunosorbent assay
GDM: gestational diabetes mellitus
GW: gestational week
HPA: hypothalamic-pituitary-adrenal
ICC: intraclass correlation coefficient
IQR: interquartile range
ITU: InTraUterine sampling in early pregnancy study
